# Association between Exposure to Traffic-Related Air Pollution and Prevalence of Allergic Diseases in Children, Seoul, Korea

**DOI:** 10.1155/2017/4216107

**Published:** 2017-09-13

**Authors:** Seon-Ju Yi, Changwoo Shon, Kyung-Duk Min, Hwan-Cheol Kim, Jong-Han Leem, Ho-Jang Kwon, Soyoung Hong, KyooSang Kim, Sun-Young Kim

**Affiliations:** ^1^Department of Public Health Science, Graduate School of Public Health, Seoul National University, Seoul, Republic of Korea; ^2^Department of Urban Society, The Seoul Institute, Seoul, Republic of Korea; ^3^Department of Occupational and Environmental Medicine, Inha University School of Medicine, Incheon, Republic of Korea; ^4^Department of Preventive Medicine, Dankook University College of Medicine, Chungnam, Republic of Korea; ^5^Department of Environmental Health Research, Seoul Medical Center, Seoul, Republic of Korea; ^6^Institute of Health and Environment, Seoul National University, Seoul, Republic of Korea; ^7^Department of Cancer Control and Population Health, Graduate School of Cancer Science and Policy, National Cancer Center, Goyang, Gyeonggi-do, Republic of Korea

## Abstract

Although there has been suggestive evidence of the association between TRAP and ADs, findings remained inconsistent possibly due to limited population. We investigated the association between TRAP and ADs in a large population of children with rich spatial coverage and expanded age span in Seoul, Korea. TRAP exposures were estimated by categorized proximity to the nearest major road (≤150, 150–300, 300–500, and >500 m) and density of major roads within 300 meters from children's residences. We estimated the association between two TRAP exposures and three ADs using generalized mixed model after adjusting for individual characteristics. We also investigated whether the association varied by household and regional socioeconomic status. We found associations of atopic eczema with road density [OR = 1.08; 95% CI = 1.01–1.15] and road proximity [1.15, 1.01–1.32; 1.17, 1.03–1.34; and 1.16, 1.01–1.34 for ≤150, 150–300, and 300–500 m, resp., compared to >500 m]. There was no association with asthma and allergic rhinitis. Effect estimates were generally the highest in the low socioeconomic region. Children living in areas surrounded by large and busy roads were likely to be at greater risks for atopic eczema, with increased vulnerability when living in deprived areas.

## 1. Introduction

Allergic diseases are the most common noncommunicable disorders of children and adolescents worldwide. Although prevalence varies by country and region, about 20–40% of children primarily suffer from symptoms of these diseases [1–3], which affect both physical and social activities of children as well as their families [4–6]. The prevalence reached a plateau or began to decrease in several countries, as understanding and management of these diseases advanced [7–10]. However, many other countries persistently showed increasing trends [11].

In addition to host risk factors for allergic diseases including genetic, behavioral, and socioeconomic components, air pollution was suggested as an environmental risk factor. In particular, recent studies focused on traffic-related air pollution (TRAP) which largely contributes to urban air pollution and possibly affects adverse health effects for large population. Epidemiologic studies reported the associations of allergic diseases for exposures to TRAP estimated by using pollutant surrogates such as nitrogen dioxide (NO_2_) and fine particulate matter (PM_2.5_) [12–15]. Other studies used direct measures of traffic including traffic volume and distance to the nearest road, focusing on traffic other than various pollutant sources, and showed inconsistent or consistent findings with those using air pollutants [15–20]. Recent toxicological studies also advanced the understanding of biological mechanism of TRAP on onset and exacerbation of allergic diseases [21]. The pathogenic pathway of TRAP on respiratory allergic diseases such as asthma had been elucidated, and the evidence of TRAP induced atopic diseases was also gradually cumulated in experimental studies and epidemiologic studies [22–24].

Despite numerous attempts to identify the causal association between TRAP exposures and allergic outcomes, particularly for nonasthmatic diseases, epidemiologic findings remained inconsistent [25]. This inconsistency might be attributed to limited study population with respect to age, space, and socioeconomic environment. First, many previous studies of TRAP and allergic diseases included children with narrow age ranges between 0 months and 17 years [25]. However, studies that assessed the effects of TRAP in children with limited age span showed only marginal associations of allergic diseases [13–15, 26]. Studies of children with limited age range may not allow us to observe phenotypes of various allergic diseases based on the natural history of atopic manifestations. Early onset of atopic eczema followed by asthma and allergic rhinitis with increased age in childhood were reported in many previous studies [27]. Second, studies were not based on the population recruited by spatial sampling [25] and their limited spatial coverage may not provide sufficient spatial heterogeneity of traffic exposures across the study areas. Furthermore, some studies reported that children in the lower socioeconomic status (SES), in both individual and regional conditions, experienced higher exposure to air pollution and larger impact on health than their counterpart in the higher SES [28, 29]. Magnitude and significance of the association may depend on diverse socioeconomic background of both household and residential area.

The Seoul Atopy Friendly School Project provided a unique opportunity to investigate the association between TRAP exposures and allergic outcomes. The city of Seoul in South Korea initiated this project to assess the prevalence and risk factors of allergic diseases in children residing in Seoul. The project recruited more than 30,000 children aged 0 to 13 and collected information on demographic characteristics, risk factors, and allergic outcomes including their home and school addresses. Seoul, the capital of South Korea, is one of the densely populated metropolitan cities with ten million people in 605 km^2^. The city reported high air pollution (PM_2.5_ annual average concentrations of 25 *μ*g/m^3^ in 2010) [30] possibly affected by heavy traffic on dense road networks. Using the Seoul Atopy Friendly School Project survey in 2010, the purpose of this study is to assess the association between exposure to TRAP and prevalence of allergic diseases. Furthermore, we investigated whether the association is modified by household and regional SES of children.

## 2. Data and Methods

### 2.1. Study Population

We obtained the Seoul Atopy Friendly School Project survey data in 2010 for 31,576 children after deidentification from the Seoul Medical Center in Seoul, Korea. Details of the survey have been described previously [31]. This cross-sectional survey recruited children from 170 schools including 136 elementary schools and 34 children's daycare centers to cover all 25 districts in Seoul.

**Figure 1:**
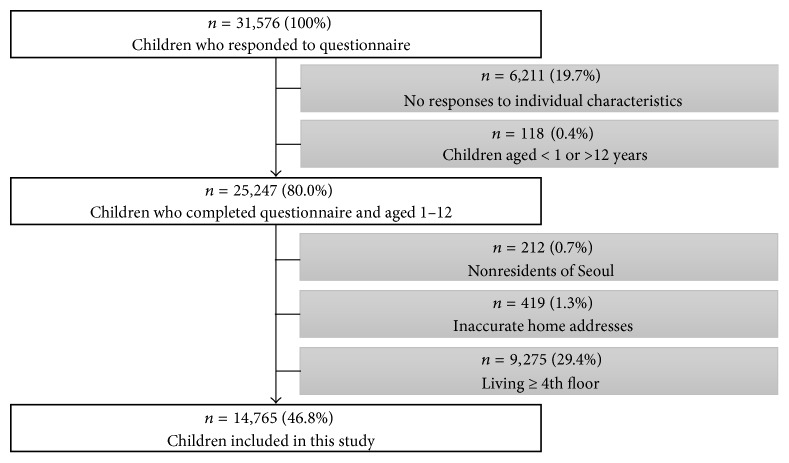
Schematic diagram of the study population selected for the present analysis using Seoul Atopy Friendly School Project survey in 2010 in Seoul, Korea.

From 31,576 children, we excluded those who did not meet our inclusion criteria (Figure 1). The excluded children did not complete questionnaire (*N* = 6,211, 19.7%), were aged less than 1 year or older than 12 years (118, 0.4%), did not live in Seoul (212, 0.7%), and had inaccurate addresses (419, 1.3%). Thirty-nine percent of children in the Seoul Atopy Friendly Project survey lived on the third floor or higher with 15% living even higher than the tenth floor. The average height of a story in multidwelling units (MDU) is about 2.8 m in Seoul [32]. Since the concentration of air pollutants emitted from roads possibly decreases as building height increases [33], we excluded children living on the 4th floor (height of about 8 m from the ground) or higher (9,275, 38.6%). These exclusions resulted in 14,765 (46.8%) children for our analysis.

### 2.2. Questionnaire Data

The questionnaire consisted of two main items: (1) sociodemographic and physical characteristics including daycare-center or school, residential address, sex, age, height, weight, household monthly income, and history of breastfeeding and (2) allergic symptoms related to atopic eczema, asthma, allergic rhinitis, and food allergy based on the modified International Study of Asthma and Allergies in Childhood (ISAAC) questionnaire. The parents or guardians completed the written questionnaire.

#### 2.2.1. Prevalence of Allergic Diseases

Prevalence of allergic diseases was asked in three ways: (1) current symptom, (2) lifetime physician diagnose, and (3) current treatment. Since healthcare utilization depended on various factors that may confound the effect of TRAP exposure [34], we used prevalence of current symptoms, as main outcomes, for three allergic diseases including atopic eczema, asthma, and allergic rhinitis. The current symptoms were defined as “symptoms in the past 12 months,” indicating itchy rash, wheezing or whistling in the chest, sneezing or runny or blocked nose without a cold or flu for atopic eczema, asthma, and allergic rhinitis, respectively.

#### 2.2.2. Assessment of Risk Factors

**Figure 2:**
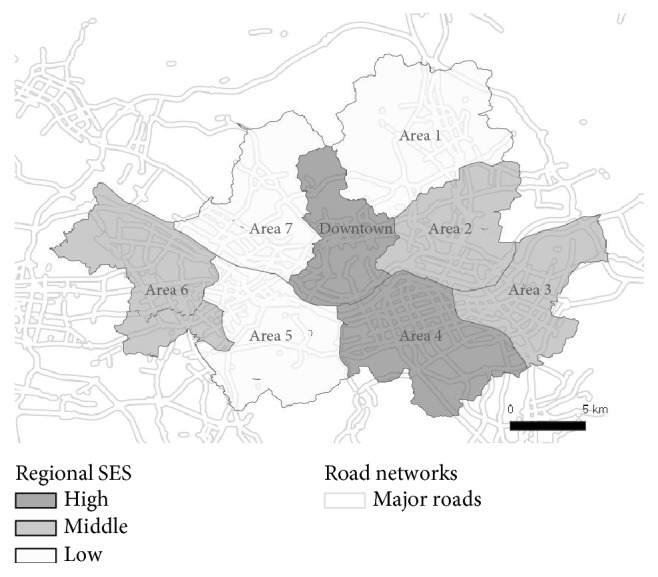
Map of eight residential areas and major roads defined as highways and roads with more than six lanes in Seoul.

For basic sociodemographic and physical information, we created categorized variables. Continuous age was classified into four groups including 1–3, 4–6, 7–9, and 10–12 years. Body mass index (BMI) was calculated as weight (in kilogram) divided by squared height (in meter) by using height and weight. Then, BMI was classified into three groups of underweight (≤25 percentile), normal (25–85), and overweight or obese (≥85) based on BMI-for-age percentiles of the 2007 Korean growth charts developed by the Korea Center for Disease Control and Prevention in 2007 [35]. Monthly household income was grouped into low (<1,720 USD), middle (1,720–3,440), and high household SES (≥3,440). Since more than half of the mothers in South Korea ceased breastfeeding in 3 months after delivery [36], we also categorized breastfeeding duration into three periods indicating never or <4 months, 4–11 months, and ≥12 months. For 25 districts in Seoul, we created eight residential areas (downtown and areas 1 to 7) combining 2–4 adjacent districts (Figure 2).

#### 2.2.3. Geocoding

We geocoded children's addresses for their homes and schools to assess traffic-related exposure, whereas we assigned coordinates of a home address to the center of a specific building; when a child resided in MDU, a school address was assigned to the center of a school boundary. Geocoding was performed by using publicly available web-based geocoding software, GeoCoder-Xr (Geoservice, Seoul, Korea).

### 2.3. Assessment of TRAP Exposure

We computed two TRAP exposure metrics including road proximity and road density for major roads based on children's home and school addresses using road network data. Maps and attributes of road networks in Seoul were obtained from the Korean Transport Database (KTDB). Road networks consist of eight classes of roads: national highways, metropolitan city highways, general national roads, metropolitan city roads, government-financed provincial roads, provincial roads, district roads, and highway link lamps. We defined major roads as national highways, metropolitan city highways, highway link lamps, and roads with more than six lanes in other five classes. Road proximity was a categorical variable derived from the continuous distance to the nearest major road and consists of four categories: ≤150 m, 150–300 m, 300–500 m, and >500 m. Road density was a continuous variable which is the sum of lengths for major roads within 300 m circular buffers. We also multiplied the road lengths by numbers of lanes and road widths to reflect traffic volume. We chose 300 m as the distance affected by traffic, as previous studies showed exponential decrease of air pollution concentrations at 300 m distant from the major roads [25, 37]. Computation procedure for distances and sums of road lengths were described in previously published work in detail [38].

Geographic data processing and variable computation were computed in ArcGIS version 10.2 (ESRI Inc., Redlands, CA, USA).

### 2.4. Statistical Analysis

Prevalence rates (PRs) of allergic diseases obtained for every stratum of individual characteristics were calculated as proportion (in percent) of children with current symptoms to the total number of children in each stratum.

We estimated odds ratios (ORs) using logistic regression to quantify the association between each pair of two TRAP exposure metrics (road proximity and density) and three allergic outcomes (atopic eczema, asthma, and allergic rhinitis). Three confounder models assessed the association after adjusting for individual- and area-level confounders and random effects. Model 1 included age and sex only, whereas Model 2 additionally included BMI, household SES, and history of breastfeeding. In Model 3, as our primary model, we added two random effect terms at school and residential area to adjust for unmeasured confounding of schools and residential areas and to account for within-school and within-area correlation of outcomes. In the main analyses, we assessed the effects of traffic exposure using home-based exposure metrics only, given the geocoding limitation of school addresses which may increase exposure measurement error [39].

We also investigated the heterogeneity of associations by children's household and regional SES using stratified analyses. Regional SES was reclassified from eight residential areas to three groups based on financial self-sufficiency proportion of revenue to expense in each district, in 2010. This district-specific financial self-sufficiency proportion was averaged for each of the eight residential areas (range = 32.5, 78.5%). Three regional SES groups included high (≥70%, downtown and area 4), middle (40–70, areas 2, 3, and 6), and low (<40, areas 1, 5, and 7) regions (Figure 2). The stratified analysis by two types of SES was performed solely and jointly. For the analysis stratified by one type of SES, we adjusted for the other type of SES.

#### 2.4.1. Sensitivity Analyses

We performed six sensitivity analyses to assess the impact of exposure measurement error and our data exclusion on the association in our primary analysis. First, we used the continuous distance instead of the categorical road proximity. To investigate the impact of misclassified traffic exposure estimates, we investigated the association in 5,211 children living on the 4th to 9th floor and 4,064 children on the 10th floor or higher. Because a previous study reported that the ISSAC questionnaire provided validated data for children aged 6-7 years and 13-14 years [11], we restricted our population to 11,803 children aged 6 or above. We also presented the results using a more conservative prevalence definition available in the questionnaire based on lifetime physician diagnosis. In our primary analysis, we excluded 3,394 children who did not report household income or breastfeeding duration which may result in the exclusion of a population subgroup with low or high socioeconomic characteristics. We assigned a new category to children with missing data for those two variables and performed analyses using 18,159 children. Lastly, we used a combined exposure metric based on home and school addresses. To reflect children's activities during school hours for approximately 8 hours, we computed average traffic exposure estimates weighted by homes twice as much as schools. Road proximity was calculated by using harmonic mean, whereas road density was computed as arithmetic mean.

The mixed effect logistic models were implemented using* lme4* package in R software version 3.3.2 (R Development Core Team, Vienna, Austria).

### 2.5. Ethical Approval

The study was approved by the institutional review board (IRB) at Seoul National University (IRB approval number: SNU IRB No. E1503/002-004).

## 3. Results

### 3.1. General Characteristics, TRAP Exposures, and Prevalence of Allergic Diseases

Table 1 shows the distribution of TRAP exposures and individual characteristics of the 14,765 children included in this analysis from the Seoul Atopy Friendly School Project survey. These children included 50% males and 26% preschoolers aged less than 6 years. Five percent of the children were overweight or obese, and more than half had breastfeeding duration less than 4 months. Eighteen percent were classified into the low household SES, while 34% lived in the low SES area. For TRAP exposures, 30, 26, and 22% of children lived at a distance within 0–150, 150–300, and 300–500 m from major roads, respectively. The mean of road density within 300 m from children's homes was 7,200 m^2^ (SD = 8,600, interquartile range (IQR) = 13,120). Out of the 170 schools, 54% were located within 300 m from major roads, and the average road density was 7,200 m^2^ (SD = 8,500, IQR = 11,430). Both road proximity and density were high in the high regional SES but similar across low to high household SES (Table S1 in Supplementary Material available online at https://doi.org/10.1155/2017/4216107).

PRs for three allergic diseases were 15.9, 8.0, and 36.2% for atopic eczema, asthma, and allergic rhinitis, respectively (Table 1). PRs for individual and socioeconomic characteristics varied by three diseases. Atopic eczema was more prevalent in girls, 4–6 years of age, the normal BMI group, and children breastfed for more than 12 months. Children with asthma symptoms were more likely to be boys, 1–3 years of age, overweight, or breastfed for more than 12 months. Allergic rhinitis was also more prevalent in boys, but these children were older, 7–9 years, underweight, or breastfed for less than 4 months. PRs for atopic eczema and allergic rhinitis were slightly lower or higher in children aged 6–12 years (PR = 15.4, 95% confidence interval (CI) 14.7–16.0; 37.3, 36.4–38.1) than those of all ages (15.9, 15.3–16.5; 36.2, 35.4–36.9), whereas PR for asthma was significantly lower (6.4, 6.0–6.9) than those for all children (8.0, 7.6–8.5). For household and regional SES, PRs for atopic eczema and asthma were high in the low and high household SES, respectively. In contrast, allergic rhinitis showed high PRs in the low regional or the high household SES (Table 1). In the joint distribution of household and regional SES, PRs for atopic eczema, asthma, and allergic rhinitis were the highest in the middle household and high regional SES, the low household and middle regional SES, and the high household and low regional SES, respectively (Table S2).

### 3.2. Associations of TRAP Exposures and Allergic Diseases

For the three allergic diseases, we found an association of atopic eczema prevalence with both traffic exposure indicators (Table 2). In Model 1, adjusting for age and sex, OR of atopic eczema for an IQR increase in the sum of major road lengths within 300 m from children's homes was 1.08 (95% CI = 1.02–1.16). This association was consistent when we added individual characteristics in Model 2 (OR = 1.08, 95% CI = 1.01–1.15) and random effects in Model 3 (1.08, 1.01–1.15). Likewise, ORs for distances to the major road of ≤150 m 150–300, and 300–500 m were significantly higher than the distance > 500 m in Model 3 (1.15, 1.01–1.32; 1.17, 1.03–1.34; 1.16, 1.01–1.34). We did not find associations of asthma and allergic rhinitis.

**Figure 3:**
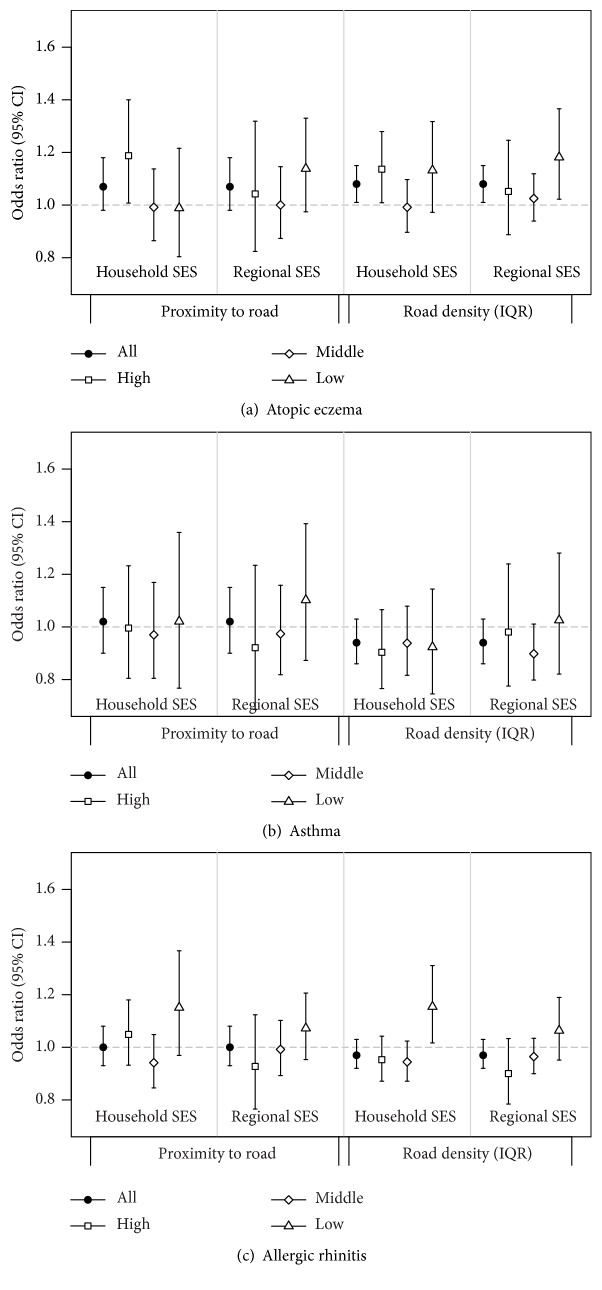
Associations between two TRAP exposures and three prevalent allergic diseases by household and regional SES in 14,765 children from the Seoul Atopy Friendly School Project Survey in 2010 in Seoul, Korea.

Figure 3 shows the associations between two TRAP exposures and three allergic diseases by household and regional SES of children. For atopic eczema, OR for an IQR increment of road density was high in the low regional SES (1.18, 1.02–1.37) and the high household SES (1.14, 1.01–1.28). In contrast, OR of allergic rhinitis was the highest in the low household SES (1.15, 1.02–1.31). There was no clear pattern for asthma (Tables S3 and S4). In a two-way stratification, OR of atopic eczema for road density (1.31, 1.04–1.66) was the highest in the high household and low regional SES, whereas OR of allergic rhinitis (1.49, 1.12–1.98) was the highest in the low household and low regional SES (Table S5).

In our sensitivity analysis, continuous distance instead of categorized distance gave consistent findings of the association with atopic eczema and no association with asthma and allergic rhinitis (Table S6). When we restricted our analysis to children living on the fourth floor or higher, the association of traffic exposure and atopic eczema disappeared. However, we found the association of road proximity with asthma and allergic rhinitis in children living on the 10th floor or higher (Table S7). The association was consistent with larger ORs for both of road proximity and density, when we added those who did not report household income and breastfeeding duration (Table S8). Another sensitivity analysis for older children and different definition of allergic diseases also showed consistent results. In the analysis for 11,803 children aged 6 or over, we found the consistent associations with larger ORs for atopic eczema for both road proximity and density (Table S9). Lifetime physician-diagnosed atopic eczema was also associated with road density and road proximity less than 300 m but with lower ORs for road proximity (Table S10). Incorporation of TRAP exposure at schools in addition to homes showed the association for road proximity with wider confidence intervals and no association for road density (Table S11).

## 4. Discussion

We examined the association of three allergic outcomes for two TRAP exposures estimated by proximity and density of major roads based on children's residences and compared the associations across three household and regional SES in a large population of children aged 1 to 12 residing in a densely populated metropolitan city. Both road density and proximity were associated with atopic eczema, whereas no association was found with asthma and allergic rhinitis. These associations were generally stronger in children living in the lower SES region.

We used proximity to the closest major road and density of nearby roads, as proxies for exposure to TRAP, to assess the associations between TRAP exposure and allergic diseases of children. Although many previous epidemiological studies of allergic diseases used exposures to traffic-related air pollutants such as PM_2.5_ and NO_X_, other studies also reported relationships using traffic indicators [25, 40–44]. These metrics help us focus on air pollution directly related to traffic, whereas it is difficult to isolate the impact of traffic when we use individual pollutants affected by various sources other than traffic [25, 45]. Some studies reported even stronger associations using traffic indicators than those of air pollution concentrations predicted by exposure prediction models such as land use regression and dispersion models [15, 46]. Although other studies raised possible exposure misclassification of proximity models [40, 47], there have also been concerns about inconsistency in monitor-based air pollution estimates when monitoring networks are sparse [48].

We used proximity and density of major roads based on road networks to represent traffic volume. Whereas previous studies of TRAP and allergic diseases mostly used proximity, we added road density which showed stronger associations than those of road proximity in our results. In addition, we incorporated numbers of lanes and line widths to road density instead of sum of single line lengths. The improved representation of the amount of traffic for road density possibly resulted in stronger associations than those for road proximity. Daily traffic volume on the roads with six lanes or more (mean = 84,310, SD = 30,744), defined as our major roads along with highways, was much higher than on the roads having less than six lanes (42,584, 17,105) at 56 traffic monitoring sites operated by the Seoul Transport Operation and Information Service [49]. In addition, air pollution concentrations measured at regulatory monitoring sites adjacent to the roads with six lanes or more were higher than concentrations at urban background monitoring sites in Seoul. The annual average concentrations of NO_2_ and PM_10_ in 2010 at urban roadside sites in Seoul (52 ppb, and 55.50 *μ*g/m^3^, resp.) were much higher than those at urban background sites (34 ppb, and 48.96 *μ*g/m^3^, resp.) [50].

We found stronger associations with atopic eczema using improved TRAP estimates with reduced exposure measurement error. Children living within the same distance to large roads may be exposed to different levels of TRAP depending on the vertical height of residences. The population affected by building heights would be large particularly in dense metropolitan areas where many people reside in MDU. In Seoul, 58% of households lived in MDU based on the 2010 population census. Smaller ORs of atopic eczema for children living on the 4th floor or higher than those for children living on the low floors, observed in our study, possibly indicate the impact of exposure measurement error on the attenuation of effect estimates. However, ORs of asthma and allergic rhinitis were higher in children living on the 4th floor or higher. Moreover, there were associations in children living on the 10th floor or higher and within 150–500 m from the closest major road. This unexpected pattern could be explained by vertical dispersion of pollutant flow disrupted by nearby buildings with downwind. Other explanations could include indoor pollutants such as semivolatile polycyclic aromatic hydrocarbon and/or different socioeconomic conditions of high floor residence [33, 51, 52]. Our study population who had large spatial coverage based on their residences and included the age range of 1 to 12 years possibly increased our ability to detect the association. The Atopy School Friendly Project survey sampled more than 30,000 children from all 25 districts of Seoul, who may represent the population of children in Seoul. This rich sample might help assess fine-scale spatial variability of exposure to traffic. The wide age range along with availability of accurate address information could have provided diversity of allergic outcomes varying by age.

We found the association of TRAP with atopic eczema but no associations with asthma and allergic rhinitis. Although all three allergic diseases, examined in this study, had similar biological mechanisms for TRAP through their immune responses [53], there were a few studies focusing on the association with nonasthmatic allergic diseases, such as atopic eczema [13, 15, 17, 26, 54–56]. A cohort study in Munich, Germany, found that road proximity, defined by 50 m to the closest major road, was associated with eczema prevalence in children aged 6 [15]. However, another German study in a different city using similar study designs and exposure assessment approaches provided different findings [20]. This inconsistency could be due to environments of study areas, children's age ranges, or limitation in exposure assessment. No associations of asthma, often reported for their associations in previous studies, also could be driven by misclassification. PR of asthma in this study based on the ISAAC questionnaire (8.0%) was higher than those in other countries, although this PR was similar to those in South Korea based on physician-diagnosed prevalence in the Korea Youth Risk Behavior Web-Based Survey and audio-visual questionnaire [57–59]. However, PR for children aged 6 or over (6.4%) was similar to PRs based on current symptoms reported in the ISAAC questionnaire in other countries (5.8 and 8.7 for ages 6-7 and 13-14, resp.) [11]. To reduce the impact of misclassified responses, we restricted our analysis to the children aged 6–12 (*N* = 11,803) in our sensitivity analysis. PR of asthma was significantly lower (PR = 6.4, 95% CI = 6.0–6.9) than PR for all children (8.0, 7.6–8.5), different from atopic eczema and allergic rhinitis showing slightly lower or higher prevalence. In contrast, ORs of TRAP were consistent between two groups of children.

A suggested biological mechanism for the association of TRAP and atopic eczema was predisposing skin barrier dysfunction followed by direct exposure of pollutants on skin. Previous toxicological studies showed that aryl hydrocarbon receptor (AhR) in cytosol of keratinocytes bound polycyclic aromatic hydrocarbons among diesel exhaust particles and activated the skin barrier dysfunction. Upon chemical binding, AhR may translocate into nuclei of a cell and induce the transcription of gene associated with generation of barrier protein including filaggrin (FLG), reactive oxygen species (ROS), and other inflammatory cytokines. FLG mutation and inflammatory process activated by upregulated genes may result in atopic eczema [24, 60–63].

We found that children's regional SES modified the association of TRAP exposure on atopic eczema and allergic rhinitis after accounting for household SES. This implies that, even under the same built environment including traffic exposure, the impact on the individual's health can be affected differentially by socioeconomic background of their residential areas. In our results, ORs of atopic eczema for TRAP were higher in children with high income family than those with low income family in the same socioeconomically deprived areas with less exposure to traffic. Generally, the housing price of house nearby major roads is higher for its transportation accessibility in Seoul. Children with high income family may live close to major roads, even if they live in low SES region, and be exposed to high TRAP. Our finding of different regional effects after adjusting for individual SES suggests that the improvement of socioeconomic environments possibly driven by public health policy implementation can reduce adverse effects of TRAP on allergic diseases of children.

The findings of this study should be interpreted with the following limitations. Our cross-sectional survey is limited to explaining the causal relationship between traffic exposure and allergic diseases. In addition, this study identified allergic diseases based on parent-reported questionnaires. Responses could have been dependent on parents' awareness on allergic symptoms which may result in outcome misclassification. There also might have been response distortions. For instance, parents could respond in a socially desirable direction. Lastly, because the survey questionnaires were not primarily designed for studies of TRAP-associated allergic diseases, we did not include important confounders such as parental history of allergic diseases and environmental smoking exposure. Future cohort studies including rich information on these confounders should confirm the association between TRAP and allergic diseases.

## 5. Conclusions

Based on a large population with rich spatial coverage and wide age range, we found that children living in areas surrounded by large and busy roads were likely to be at greater risks for atopic eczema, with increased vulnerability when living in deprived areas.

## Supplementary Material

Table S1. Summary statistics of distances and road density by regional and household SES.
Table S2. Prevalence of allergic diseases by regional and household SES.
Table S3. Associations between two TRAP exposures and three allergic diseases from Model 3 by regional SES.
Table S4. Associations between two TRAP exposures and three allergic diseases from Model 3 by household SES.
Table S5. Associations between two TRAP exposures and three allergic diseases from Model 3 by regional and household SES.
Table S6. Associations between the distances and three allergic diseases.
Table S7. Associations between two TRAP exposures and three allergic diseases from Model 3 by residential floor levels in 24,040 children including children on the 4th floor or over.
Table S8. Associations between two TRAP exposures and three allergic diseases from Model 3 in 18,159 children including children who did not respond to questionnaire for individual characteristics.
Table S9. Associations between two TRAP exposures and three allergic diseases from Model 3, and disease prevalence rates (PRs) in 11,803 children aged 6-12 years.
Table S10. Associations between two TRAP exposures and lifetime physician-diagnosed allergic diseases from Model 3.
Table S11. Associations between two TRAP exposures and three allergic diseases from Model 3 using TRAP exposure estimates based on home as well as school addresses.

## Figures and Tables

**Table 1:** Summary statistics of TRAP exposures for each allergic disease and prevalence of three allergic diseases for each individual characteristic in 14,765 children from the Seoul Atopy Friendly School Project survey in 2010 in Seoul, Korea.

	Total		Prevalent cases (prevalence rate, %)					
	*N* (%)		Atopic eczema		Asthma		Allergic rhinitis	
	14,765	(100)	2,351	(15.9)	1,187	(8.0)	5,338	(36.2)
TRAP exposure^a^								
Proximity^b^								
0–150 m	**4,494**	(30.4)	**724**	(30.8)	**314**	(26.5)	**1,601**	(30.0)
150–300 m	**3,873**	(26.2)	**629**	(26.8)	**332**	(28.0)	**1,432**	(26.8)
300–500 m	**3,284**	(22.2)	**536**	(22.8)	**261**	(22.0)	**1,193**	(22.3)
>500 m	**3,114**	(21.1)	**462**	(19.7)	**280**	(23.6)	**1,112**	(20.8)
Density^c^ (1,000 m^2^)	**7.2**	(8.6)	**7.5**	(8.8)	**6.7**	(8.2)	**7.1**	(8.6)

Sex								
Girls	7,356	(49.8)	1,198	(16.3)	468	(6.4)	2,341	(31.8)
Boys	7,409	(50.2)	1,153	(15.6)	719	(9.7)	2,997	(40.5)
Age								
1–3	1,322	(9.0)	223	(16.9)	235	(17.8)	369	(27.9)
4–6	2,447	(16.6)	450	(18.4)	271	(11.1)	890	(36.4)
7–9	5,453	(36.9)	898	(16.5)	386	(7.1)	2,089	(38.3)
10–12	5,543	(37.5)	780	(14.1)	295	(5.3)	1,990	(35.9)
Body fatness								
Normal	11,948	(80.9)	1,944	(16.3)	930	(7.8)	4,214	(35.3)
Overweight or obese	769	(5.2)	122	(15.9)	89	(11.6)	283	(36.8)
Underweight	2,048	(13.9)	285	(13.9)	168	(8.2)	841	(41.1)
Breastfeeding duration								
<4 months	8,042	(54.5)	1,124	(14.0)	597	(7.4)	2,977	(37.0)
4–11	3,876	(26.3)	637	(16.4)	318	(8.2)	1,342	(34.6)
≥12	2,847	(19.3)	590	(20.7)	272	(9.6)	1,019	(35.8)
Residential area								
Downtown	1,729	(11.7)	315	(18.2)	191	(11.0)	543	(31.4)
Area 1	1,803	(12.2)	270	(15.0)	106	(5.9)	665	(36.9)
Area 2	3,010	(20.4)	552	(18.3)	366	(12.2)	1,048	(34.8)
Area 3	2,303	(15.6)	318	(13.8)	140	(6.1)	807	(35.0)
Area 4	973	(6.6)	154	(15.8)	59	(6.1)	359	(36.9)
Area 5	1,282	(8.7)	196	(15.3)	73	(5.7)	492	(38.4)
Area 6	1,678	(11.4)	246	(14.7)	122	(7.3)	639	(38.1)
Area 7	1,987	(13.5)	300	(15.1)	130	(6.5)	785	(39.5)
Household SES								
High	5,545	(37.6)	770	(13.9)	423	(7.6)	2,120	(38.2)
Middle	6,539	(44.3)	1,097	(16.8)	532	(8.1)	2,367	(36.2)
Low	2,681	(18.2)	484	(18.1)	232	(8.7)	851	(31.7)
Regional SES^d^								
High	2,702	(18.3)	469	(17.4)	250	(9.3)	902	(33.4)
Middle	6,991	(47.3)	1,116	(16.0)	628	(9.0)	2,494	(35.7)
Low	5,072	(34.4)	766	(15.1)	309	(6.1)	1,942	(38.3)

^a^Number of children (percent) for each allergic disease is displayed for proximity, whereas mean (standard deviation) is presented for summarizing road density. ^b^Proximity is a categorical variable indicating children live within a specific distance from the closest major roads. ^c^Density is a continuous variable defined as the sum of road lengths multiplied by numbers of lanes and road widths of major roads within a 300 m radius buffer from a child's home. ^d^Regional socioeconomic status (SES) was categorized based on fiscal self-sufficiency of residential areas (see Figure 2).

**Table 2:** Associations between two TRAP exposures and three allergic diseases in 14,765 children from the Seoul Atopy Friendly School Project Survey in 2010 in Seoul, Korea.

	Model 1		Model 2		Model 3	
	OR^a^	95% CI	OR^b^	95% CI	OR^c^	95% CI
*Proximity*						
Atopic eczema						
≤150 m	**1.16**	**(1.02–1.32)**	**1.15**	**(1.01–1.31)**	**1.15**	**(1.01–1.32)**
150–300 m	**1.17**	**(1.02–1.34)**	**1.17**	**(1.03–1.34)**	**1.17**	**(1.03–1.34)**
300–500 m	**1.16**	**(1.01–1.33)**	**1.16**	**(1.01–1.33)**	**1.16**	**(1.01–1.34)**
>500 m	1.00		1.00		1.00	
Asthma						
0–150 m	0.95	(0.80–1.13)	0.94	(0.79–1.12)	0.93	(0.78–1.11)
150–300 m	1.13	(0.95–1.34)	1.12	(0.95–1.33)	1.11	(0.93–1.32)
300–500 m	1.01	(0.85–1.21)	1.01	(0.84–1.21)	1.00	(0.83–1.20)
>500 m	1.00		1.00		1.00	
Allergic rhinitis						
0–150 m	0.96	(0.87–1.05)	0.96	(0.87–1.06)	0.97	(0.88–1.07)
150–300 m	1.03	(0.93–1.14)	1.03	(0.93–1.14)	1.05	(0.95–1.16)
300–500 m	0.99	(0.89–1.10)	1.00	(0.90–1.10)	1.00	(0.90–1.12)
>500 m	1.00		1.00		1.00	

*Density for an interquartile range increment (13,120 m* ^*2*^)						
Atopic eczema	**1.08 **	**(1.02–1.16)**	**1.08 **	**(1.01–1.15)**	**1.08 **	**(1.01–1.15)**
Asthma	0.95	(0.87–1.05)	0.95	(0.87–1.04)	0.94	(0.86–1.03)
Allergic rhinitis	0.97	(0.92–1.02)	0.97	(0.92–1.03)	0.97	(0.92–1.03)

^a^Odds ratio (OR) adjusted for sex and age; ^b^OR adjusted for sex, age, household monthly income, body mass index, and history of breastfeeding; ^c^OR adjusted for sex, age, household monthly income, body mass index, history of breastfeeding, and random effects for school and residential area.
