# Supplementary material for: Association between Exposure to Traffic-Related Air Pollution and Prevalence of Allergic Diseases in Children, Seoul, Korea
Source: Biomed Res Int. 2017 Sep 13;2017:4216107. doi: 10.1155/2017/4216107 (PMC5615949; doi:10.1155/2017/4216107)
Supplement: Supplementary file 1 — Table S1. Summary statistics of distances and road density by regional and household SES. Table S2. Prevalence of allergic diseases by regional and household SES. Table S3. Associations between two TRAP exposures and three allergic diseases from Model 3 by regional SES. Table S4. Associations between two TRAP exposures and three allergic diseases from Model 3 by household SES. Table S5. Associations between two TRAP exposures and three allergic diseases from Model 3 by regional and household SES. Table S6. Associations between the distances and three allergic diseases. Table S7. Associations between two TRAP exposures and three allergic diseases from Model 3 by residential floor levels in 24,040 children including children on the 4th floor or over. Table S8. Associations between two TRAP exposures and three allergic diseases from Model 3 in 18,159 children including children who did not respond to questionnaire for individual characteristics. Table S9. Associations between two TRAP exposures and three allergic diseases from Model 3, and disease prevalence rates (PRs) in 11,803 children aged 6-12 years. Table S10. Associations between two TRAP exposures and lifetime physician-diagnosed allergic diseases from Model 3. Table S11. Associations between two TRAP exposures and three allergic diseases from Model 3 using TRAP exposure estimates based on home as well as school addresses. [file 4216107.f1.pdf]

Table S1. Distribution of distance from nearest roads and road density by regional and household SES in 14,765 children from the Seoul Atopy Friendly School Project Survey during 2010 in Seoul, Korea

		N (%)		Distance (m)		Density (km^2)	
				Mean	(SD)	Mean	(SD)
**Total**		14,765	(100)	320.75	(261.60)	7.16	(8.65)
**Regional SES**	**Household SES**						
High		5,545	(37.6)	256.76	(218.57)	9.27	(8.37)
Middle		6,539	(44.3)	273.56	(220.76)	8.23	(9.50)
Low		2,681	(18.2)	419.87	(302.56)	4.57	(6.73)
	High	2,702	(18.3)	324.55	(265.80)	7.11	(8.58)
	Middle	6,991	(47.3)	319.69	(261.75)	7.25	(8.71)
	Low	5,072	(34.4)	315.45	(252.29)	7.05	(8.65)
High	High	955	(6.5)	249.69	(218.02)	9.76	(8.51)
High	Middle	1,165	(7.9)	258.55	(223.33)	9.21	(8.25)
High	Low	585	(4.0)	264.68	(209.74)	8.58	(8.17)
Middle	High	2,588	(17.5)	270.68	(216.46)	8.21	(9.33)
Middle	Middle	3,129	(21.2)	274.72	(224.68)	8.34	(9.61)
Middle	Low	1,274	(8.6)	276.57	(219.81)	8.00	(9.60)
Low	High	2,005	(13.6)	429.63	(308.15)	4.43	(6.68)
Low	Middle	2,245	(15.2)	414.10	(299.30)	4.71	(6.80)
Low	Low	822	(5.6)	411.85	(296.77)	4.49	(6.64)

Table S2. Prevalence of allergic diseases by regional and household SES in 14,765 children from the Seoul Atopy Friendly School Project Survey during 2010 in Seoul, Korea

		Atopic eczema		Asthma		Allergic rhinits	
		PR	(95% CI)	PR	(95% CI)	PR	(95% CI)
**Total**		15.9	(15.3 - 16.9)	8.0	(7.6 - 8.5)	36.2	(35.4 - 36.9)
**Regional SES**	**Household SES**						
High		17.4	(15.9 - 18.8)	9.3	(8.2 - 10.3)	33.4	(31.6 - 35.2)
Middle		16.0	(15.1 - 16.8)	9.0	(8.3 - 9.7)	35.7	(34.6 - 36.8)
Low		15.1	(14.1 - 16.1)	6.1	(5.4 - 6.8)	38.3	(37.0 - 39.6)
	High	13.9	(13.0 - 14.8)	7.6	(6.9 - 8.3)	38.2	(37.0 - 39.5)
	Middle	16.8	(15.9 - 17.7)	8.1	(7.5 - 8.8)	36.2	(35.0 - 37.4)
	Low	18.1	(16.6 - 19.5)	8.7	(7.6 - 9.7)	31.7	(30.0 - 33.5)
High	High	13.8	(11.6 - 15.9)	9.3	(7.5 - 11.2)	36.2	(33.2 - 39.3)
High	Middle	20.2	(17.9 - 22.5)	9.8	(8.1 - 11.5)	33.1	(30.4 - 35.8)
High	Low	17.6	(14.5 - 20.7)	8.0	(5.8 - 10.2)	29.2	(25.5 - 32.9)
Middle	High	14.2	(12.8 - 15.5)	8.5	(7.4 - 9.5)	36.9	(35.1 - 38.8)
Middle	Middle	16.8	(15.5 - 18.2)	8.8	(7.8 - 9.8)	36.1	(34.5 - 37.8)
Middle	Low	17.4	(15.3 - 19.5)	10.5	(8.8 - 12.2)	31.9	(29.4 - 34.5)
Low	High	13.6	(12.1 - 15.1)	5.7	(4.7 - 6.8)	40.8	(38.7 - 43.0)
Low	Middle	14.9	(13.4 - 16.4)	6.4	(5.4 - 7.4)	37.9	(35.9 - 39.9)
Low	Low	19.3	(16.6 - 22.0)	6.2	(4.6 - 7.9)	33.2	(30.0 - 36.4)

Table S3. Association between two TRAP exposures and three allergic diseases on Model 3 by regional SES in 14,765 children from the Seoul Atopy Friendly School Project Survey during 2010 in Seoul, Korea

	High SES		Middle SES		Low SES	
	**OR**	**95% C.I.**	**OR**	**95% C.I.**	**OR**	**95% C.I.**
**Proximity**						
Atopic eczema						
≤150m	1.30	(0.93 - 1.82)	0.98	(0.79 - 1.22)	**1.27**	**(1.02 - 1.57)**
150-300m	1.15	(0.81 - 1.63)	1.11	(0.89 - 1.37)	1.19	(0.96 - 1.48)
300-500m	1.33	(0.92 - 1.92)	1.06	(0.85 - 1.33)	1.19	(0.97 - 1.47)
>500m	1.00		1.00		1.00	
Asthma						
≤150m	1.01	(0.67 - 1.53)	0.84	(0.64 - 1.09)	0.93	(0.67 - 1.28)
150-300m	1.08	(0.72 - 1.62)	1.05	(0.81 - 1.37)	1.12	(0.82 - 1.52)
300-500m	1.28	(0.85 - 1.95)	0.94	(0.72 - 1.24)	0.82	(0.60 - 1.13)
>500m	1.00		1.00		1.00	
Allergic rhinitis						
≤150m	0.89	(0.68 - 1.18)	1.00	(0.84 - 1.19)	1.04	(0.89 - 1.22)
150-300m	1.06	(0.80 - 1.40)	1.07	(0.90 - 1.27)	1.07	(0.91 - 1.25)
300-500m	1.07	(0.79 - 1.45)	1.06	(0.89 - 1.27)	0.96	(0.82 - 1.12)
>500m	1.00		1.00		1.00	
**Density for an interquartile range increase (13,120m^2)**						
Atopic eczema	1.05	(0.89 - 1.25)	1.02	(0.94 - 1.12)	**1.18**	**(1.02 - 1.37)**
Asthma	0.98	(0.78 - 1.24)	0.90	(0.80 - 1.01)	1.03	(0.82 - 1.28)
Allergic rhinitis	0.90	(0.78 - 1.03)	0.96	(0.90 - 1.03)	1.06	(0.95 - 1.19)

Table S4. Association between two TRAP exposures and three allergic diseases on Model 3 by household SES in 14,765 children from the Seoul Atopy Friendly School Project Survey during 2010 in Seoul, Korea

	**High SES**		**Middle SES**		**Low SES**	
	**OR**	**95% C.I.**	**OR**	**95% C.I.**	**OR**	**95% C.I.**
**Proximity**						
Atopic eczema						
≤150m	**1.32**	**(1.05 - 1.67)**	0.98	(0.81 - 1.19)	1.21	(0.89 - 1.65)
150-300m	1.19	(0.94 - 1.52)	1.12	(0.92 - 1.37)	1.17	(0.86 - 1.59)
300-500m	1.11	(0.87 - 1.42)	1.10	(0.90 - 1.35)	1.38	(1.02 - 1.88)
>500m	1.00		1.00		1.00	
Asthma						
≤150m	0.92	(0.68 - 1.25)	0.90	(0.69 - 1.18)	0.78	(0.52 - 1.17)
150-300m	1.15	(0.85 - 1.55)	1.09	(0.84 - 1.41)	0.92	(0.62 - 1.36)
300-500m	1.06	(0.78 - 1.44)	1.04	(0.79 - 1.36)	0.70	(0.46 - 1.07)
>500m	1.00		1.00		1.00	
Allergic rhinitis						
≤150m	1.06	(0.90 - 1.26)	0.89	(0.76 - 1.03)	1.13	(0.88 - 1.45)
150-300m	1.17	(0.98 - 1.38)	0.96	(0.82 - 1.12)	1.15	(0.90 - 1.48)
300-500m	1.11	(0.93 - 1.31)	0.96	(0.82 - 1.12)	0.98	(0.76 - 1.27)
>500m	1.00		1.00		1.00	
**Density for an interquartile range increase (13,120m^2)**						
Atopic eczema	**1.14**	**(1.01 - 1.28)**	0.99	(0.90 - 1.10)	1.13	(0.97 - 1.32)
Asthma	0.90	(0.77 - 1.07)	0.94	(0.82 - 1.08)	0.92	(0.75 - 1.14)
Allergic rhinitis	0.95	(0.87 - 1.04)	0.94	(0.87 - 1.02)	**1.15**	**(1.02 - 1.31)**

Table S5. Association between two TRAP exposures and three allergic diseases on Model 3 by joint combinations of regional and household SES in 14,765 children from the Seoul Atopy Friendly School Project Survey during 2010 in Seoul, Korea

		Atopic eczema	Asthma	Allergic rhinitis
		OR (95% CI)	OR (95% CI)	OR (95% CI)
**Proximity (≤300m)**				
**Regional SES**	**Household SES**			
High	High	0.94 (0.60 - 1.47)	0.80 (0.48 - 1.33)	0.78 (0.56 - 1.03)
High	Middle	1.14 (0.81 - 1.60)	0.90 (0.58 - 1.39)	0.92 (0.69 - 1.23)
High	Low	0.95 (0.58 - 1.57)	1.06 (0.54 - 2.07)	1.27 (0.83 - 1.94)
Middle	High	1.18 (0.93 - 1.51)	1.00 (0.74 - 1.35)	1.06 (0.89 - 1.27)
Middle	Middle	0.90 (0.74 - 1.10)	0.95 (0.73 - 1.23)	0.92 (0.79 - 1.07)
Middle	Low	0.97 (0.71 - 1.32)	1.01 (0.69 - 1.48)	1.00 (0.78 - 1.29)
Low	High	1.27 (0.98 - 1.65)	1.11 (0.75 - 1.63)	1.11 (0.92 - 1.33)
Low	Middle	1.10 (0.87 - 1.39)	1.11 (0.79 - 1.57)	0.98 (0.82 - 1.17)
Low	Low	1.03 (0.72 - 1.46)	1.16 (0.65 - 2.08)	1.28 (0.95 - 1.72)
**Density for an interquartile range increment (13,120m^2)**				
**Regional SES**	**Household SES**			
High	High	0.99 (0.72 - 1.36)	0.91 (0.61 - 1.36)	0.81 (0.64 - 1.02)
High	Middle	1.10 (0.87 - 1.40)	0.99 (0.70 - 1.41)	0.87 (0.71 - 1.07)
High	Low	1.01 (0.69 - 1.47)	0.99 (0.57 - 1.71)	1.17 (0.86 - 1.60)
Middle	High	1.10 (0.94 - 1.28)	0.86 (0.70 - 1.06)	0.94 (0.83 - 1.06)
Middle	Middle	0.92 (0.81 - 1.05)	0.92 (0.78 - 1.10)	0.95 (0.86 - 1.06)
Middle	Low	1.18 (0.97 - 1.44)	0.90 (0.70 - 1.17)	1.03 (0.88 - 1.22)
Low	High	**1.31 (1.04 - 1.66)**	1.06 (0.73 - 1.53)	1.05 (0.88 - 1.26)
Low	Middle	1.14 (0.92 - 1.38)	1.00 (0.72 - 1.38)	0.96 (0.81 - 1.14)
Low	Low	1.08 (0.77 - 1.52)	1.09 (0.63 - 1.86)	**1.49 (1.12 - 1.98)**

Table S6. Association between the distance from the closest major roads and three allergic diseases in 14,765 children from the Seoul Atopy Friendly School Project Survey during 2010 in Seoul, Korea

	**Model 1**		**Model 2**		**Model 3**	
	**OR[a]**	**95% C.I.**	**OR[b]**	**95% C.I.**	**OR[c]**	**95% C.I.**
**Distance to nearest major roads for 300 meters increment**						
Atopic eczema	**0.93**	**(0.88 - 0.98)**	**0.95**	**(0.90 - 1.00)**	**0.93**	**(0.89 - 0.99)**
Asthma	1.00	(0.93 - 1.07)	1.03	(0.96 - 1.11)	1.01	(0.94 - 1.09)
Allergic rhinitis	1.01	(0.97 - 1.05)	1.00	(0.96 - 1.04)	1.01	(0.96 - 1.05)

a. Odds ratio (OR) adjusted for sex and age; b. OR adjusted for sex, age, household monthly income, body mass index and history of breastfeeding; c. OR adjusted for sex, age, household monthly income, body mass index and history of breastfeeding, random effects for school and residential area

Table S7. Association between two TRAP exposures and three allergic diseases on Model 3 by residential floor levels in 24,040 children including 5,211 children living on the 4th to 9th floor and 4,064 children on the 10th floor or over from the Seoul Atopy Friendly School Project Survey during 2010 in Seoul, Korea

	**All floors (N=24,040)**		**4-9th floors (N=5,211)**		**≥10 floors (N=4,064)**	
	**OR**	**95% C.I.**	**OR**	**95% C.I.**	**OR**	**95% C.I.**
**Proximity**						
Atopic eczema						
≤150m	1.04	(0.94 - 1.15)	0.81	(0.63 - 1.03)	0.86	(0.65 - 1.14)
150-300m	1.11	(0.99 - 1.23)	0.85	(0.66 - 1.10)	1.08	(0.80 - 1.45)
300-500m	1.10	(0.98 - 1.23)	0.86	(0.65 - 1.13)	1.07	(0.78 - 1.48)
>500m	1.00		1.00		1.00	
Asthma						
≤150m	1.01	(0.87 - 1.16)	1.15	(0.81 - 1.62)	1.48	(0.95 - 2.31)
150-300m	**1.17**	**(1.01 - 1.35)**	1.19	(0.83 - 1.69)	**1.86**	**(1.18 - 2.94)**
300-500m	1.12	(0.96 - 1.31)	1.36	(0.94 - 1.97)	**1.87**	**(1.15 - 3.04)**
>500m	1.00		1.00		1.00	
Allergic rhinitis						
≤150m	1.01	(0.93 - 1.10)	1.00	(0.82 - 1.21)	1.22	(0.97 - 1.53)
150-300m	1.07	(0.98 - 1.16)	0.98	(0.80 - 1.19)	**1.34**	**(1.05 - 1.70)**
300-500m	1.05	(0.96 - 1.15)	1.11	(0.90 - 1.37)	1.18	(0.91 - 1.54)
>500m	1.00		1.00		1.00	
**Density for an interquartile range increment (13,120m²)**						
Atopic eczema	1.03	(0.97 - 1.08)	0.93	(0.83 - 1.05)	0.94	(0.83 - 1.07)
Asthma	0.96	(0.89 - 1.03)	0.97	(0.83 - 1.13)	1.01	(0.85 - 1.20)
Allergic rhinitis	0.99	(0.95 - 1.03)	0.96	(0.88 - 1.05)	1.04	(0.95 - 1.15)

Table S8. Association between two TRAP exposures and three allergic diseases on Model 3 in 18,159 children including 14,765 in our primary analysis and 3,394 who did not respond to questionnaire for individual characteristics from the Seoul Atopy Friendly School Project Survey during 2010 in Seoul, Korea

	**Atopic eczema**		**Asthma**		**Allergic rhinitis**	
	**OR**	**95% C.I.**	**OR**	**95% C.I.**	**OR**	**95% C.I.**
**Proximity**						
≤150m	**1.21**	**(1.07 - 1.36)**	1.01	(0.86 - 1.18)	0.97	(0.89 - 1.06)
150-300m	**1.22**	**(1.08 - 1.38)**	1.11	(0.95 - 1.30)	1.05	(0.96 - 1.15)
300-500m	**1.18**	**(1.04 - 1.34)**	1.05	(0.89 - 1.24)	1.02	(0.92 - 1.12)
>500m	1.00		1.00		1.00	
**Density for an interquartile range increment (13,120m²)**						
	**1.09**	**(1.02 - 1.15)**	0.97	(0.89 - 1.05)	0.98	(0.93 - 1.02)

Table S9. Association between two TRAP exposures and three allergic diseases on Model 3 and disease prevalence rates (PRs) in 11,803 children aged 6-12 years from the Seoul Atopy Friendly School Project Survey during 2010 in Seoul, Korea

	Atopic eczema		Asthma		Allergic rhinitis	
**Odds ratios (ORs)**	**OR**	**95% C.I.**	**OR**	**95% C.I.**	**OR**	**95% C.I.**
**Proximity**						
≤150m	**1.28**	**(1.09 - 1.49)**	0.98	(0.78 - 1.23)	0.98	(0.87 - 1.10)
150-300m	**1.34**	**(1.14 - 1.57)**	1.16	(0.92 - 1.46)	1.06	(0.94 - 1.19)
300-500m	**1.24**	**(1.05 - 1.46)**	1.06	(0.84 - 1.35)	1.03	(0.91 - 1.16)
>500m	1.00		1.00		1.00	
**Density for an interquartile range increment (13,120m^2)**						
	**1.12**	**(1.03 - 1.21)**	0.94	(0.83 - 1.06)	0.97	(0.91 - 1.03)
**Prevalence rates (PRs)**	**PR**	**95% C.I.**	**PR**	**95% C.I.**	**PR**	**95% C.I.**
All ages (N=14,765)	15.9	(15.3 - 16.9)	8.0	(7.6 - 8.5)	36.2	(35.4 - 36.9)
Aged 6-12 years (N=11,803)	15.4	(14.7 - 16.0)	6.4	(6.0 - 6.9)	37.3	(36.4 - 38.1)

Table S10. Association between two TRAP exposures and lifetime physician-diagnosed allergic diseases on Model 3 in 14,765 children from the Seoul Atopy Friendly School Project Survey during 2010 in Seoul, Korea

	Atopic eczema		Asthma		Allergic rhinitis	
**Odds ratios (ORs)**	**OR**	**95% C.I.**	**OR**	**95% C.I.**	**OR**	**95% C.I.**
**Proximity**						
≤150m	**1.11**	**(1.01 - 1.23)**	0.98	(0.82 - 1.18)	1.02	(0.91 - 1.14)
150-300m	**1.11**	**(1.00 - 1.23)**	0.96	(0.80 - 1.16)	1.01	(0.91 - 1.13)
300-500m	1.04	(0.94 - 1.16)	1.02	(0.84 - 1.23)	1.06	(0.95 - 1.19)
>500m	1.00		1.00		1.00	
**Density for an interquartile range increment (13,120m^2)**						
	**1.10**	**(1.04 - 1.15)**	0.88	(0.80 - 0.97)	0.99	(0.93 - 1.04)
**Prevalence rates (PRs)**	**PR**	**95% C.I.**	**PR**	**95% C.I.**	**PR**	**95% C.I.**
All ages (N=14,765)	15.9	(15.3 - 16.9)	8.0	(7.6 - 8.5)	36.2	(35.4 - 36.9)

Table S11. Association between two TRAP exposures, estimated based on schools in addition to residences, and three allergic diseases on Model 3 in 14,765 children from the Seoul Atopy Friendly School Project Survey during 2010 in Seoul, Korea

	Atopic eczema		Asthma		Allergic rhinitis	
	**OR**	**95% C.I.**	**OR**	**95% C.I.**	**OR**	**95% C.I.**
**Proximity**						
≤150m	**1.20**	**(1.02 - 1.43)**	0.98	(0.79 - 1.21)	0.92	(0.80 - 1.05)
150-300m	**1.18**	**(1.00 - 1.41)**	0.97	(0.78 - 1.21)	0.98	(0.86 - 1.11)
300-500m	1.17	(0.98 - 1.40)	0.97	(0.77 - 1.22)	0.99	(0.87 - 1.13)
>500m	1.00		1.00		1.00	
**Density for an interquartile range increment (9,164m^2)**						
	1.05	(0.99 - 1.13)	0.94	(0.86 - 1.03)	0.96	(0.91 - 1.02)
